# Spontaneous rupture of splenic artery pseudoaneurysm

**DOI:** 10.1093/jscr/rjac604

**Published:** 2022-12-30

**Authors:** Andriy Hordiychuk, Daniel Mehanna

**Affiliations:** Department of Surgery, Caboolture Hospital, QLD Health, QLD, Australia; School of Clinical Medicine, University of Queensland, QLD, Australia; Department of Surgery, Caboolture Hospital, QLD Health, QLD, Australia

## Abstract

False aneurysm or visceral artery pseudoaneurysm (VAPA) results from a tear in the vessel wall due to trauma with subsequent periarterial haematoma formation. VAPA is relatively rare, with a reported incidence of 0.1–0.2%, although the actual incidence is not known since many are asymptomatic. Splenic artery pseudoaneurysm is even more rare pathology. Only around 200 cases have been described in the literature. The case report below describes a spontaneous rupture of splenic artery pseudoaneurysm.

## INTRODUCTION

False aneurysm or visceral artery pseudoaneurysm (VAPA) results from a tear in the vessel wall due to trauma or pancreatitis with subsequent periarterial hematoma formation [1]. VAPA is relatively rare, with a reported incidence of 0.1–0.2%, although the actual incidence is not known since many are asymptomatic. Splenic artery pseudoaneurysm (SAP) is even more rare pathology. Only around 200 cases have been described in the literature [2].

## CASE PRESENTATION

A 66-year-old female presented to the hospital with sudden onset of severe epigastric pain that irradiated to the back, nausea and vomiting and pre-syncope while sitting on a couch and watching TV in the evening. There were no recent reported falls or any other trauma.

The patient has a medical history of mechanical aortic and mitral valve repair decades ago (on Warfarin), hypertension and spontaneous abdominal wall haematoma evacuation in theatre. Also, she had a fall from a standing height and landed on her left side of the chest and abdomen 1 year prior to this hospital presentation.

The patient was pale, diaphoretic and tachycardic up to 102 bpm (beats per minute) and had a blood pressure of 108/82 mmHg on arrival to the emergency department (ED). Her Hb (haemoglobin) level was 112, and INR 2.6 (international normalized ratio) was within the therapeutic range for warfarin therapy. The patient was stable enough to proceed with radiological imaging as the next step of a work-up.

**Figure 1:**
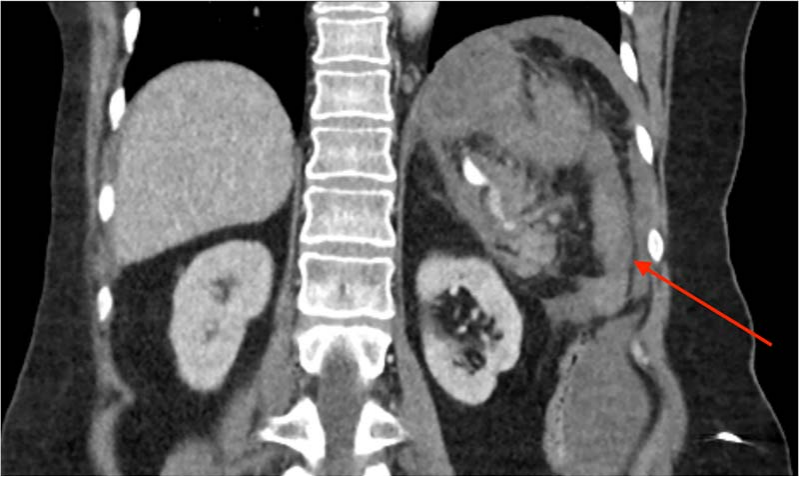
CT of the abdomen and pelvis, coronal plane, arterial phase. Large intraabdominal haematoma in the left upper quadrant.

**Figure 2:**
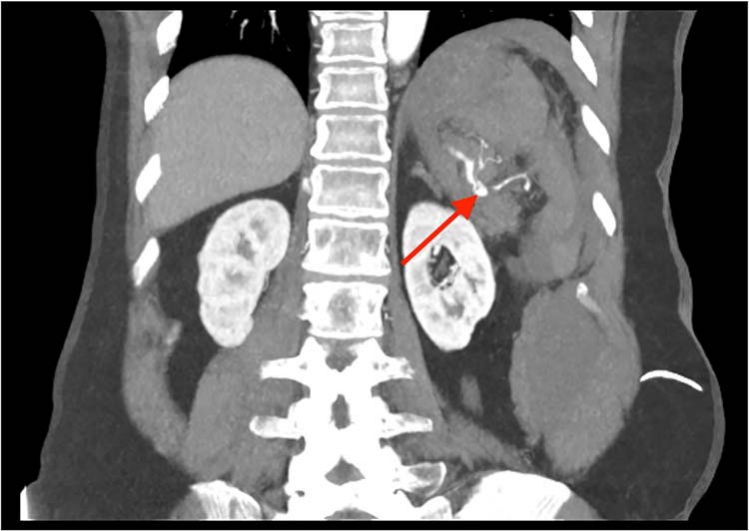
CT of the abdomen and pelvis, coronal plane, arterial phase. Contrast extravasation from the ruptured SAP.

**Figure 3:**
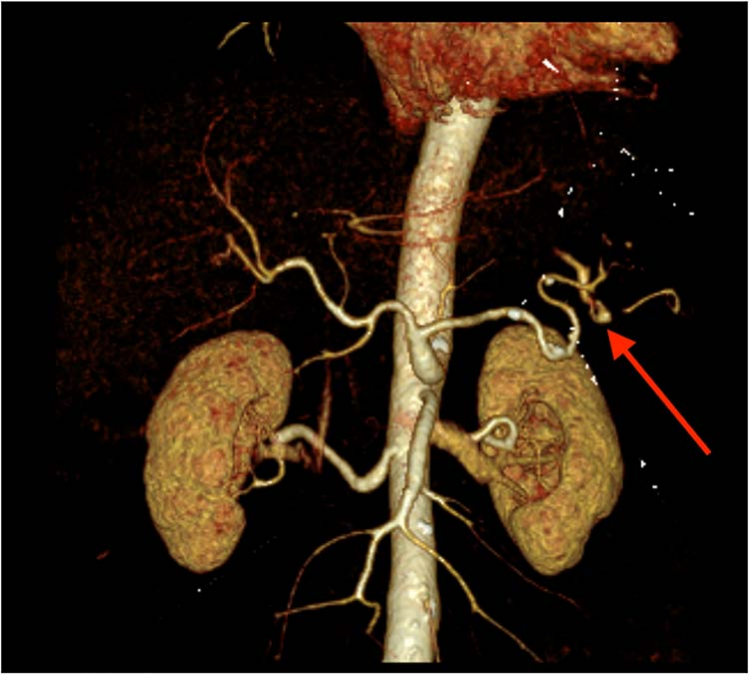
CT angiography of the abdomen with 3D reconstruction. Ruptured SAP.

Triple-phase computed tomography (CT) of the abdomen and pelvis revealed a sizeable acute haematoma in the left upper abdomen, below the gastric fundus and body, alongside the pancreatic tail and splenic hilum, extending amongst the proximal small bowel mesentery and into the left subphrenic space. The origin of the haemorrhage appeared to arise from the left upper quadrant. There was a pseudoaneurysm in close proximity to the pancreatic tail and splenic hilum, apparently arising from the adjacent distal splenic artery. CT scan did show local contrast extravasation into the haematoma, as mentioned above (Figs 1–3).

Administration of intravenous vitamin K and Prothrombinex-VF were commenced for warfarin reversal as per guideline. Upon returning from CT to the ED, the patient started to deteriorate and developed a hypovolaemic shock despite ongoing resuscitation with intravenous fluids. Intravenous vasopressors were started.

The patient was haemodynamically unstable for inter-hospital transfer to a tertiary hospital for consideration of embolization of the splenic artery and was urgently taken to theatre for exploratory laparotomy. A large haemoperitoneum was found intraoperatively. A splenectomy was performed due to an unsuccessful attempt to control the bleeding from the ruptured aneurysm of splenic artery.

The patient recovered well after the splenectomy. Surgical outpatient follow-up performed in 3-, 5- and 7- weeks post-surgery. Also, the patient underwent recommended vaccinations as per post-splenectomy protocol – Pneumococcal, Meningococcal and *Haemophilus influenzae* type B with scheduled annual influenza vaccination.

## DISCUSSION

VAPA represents a confined rupture that is only constrained by a fibrous capsule because the layers of the arterial wall are disrupted (usually two of them – intima and media) in comparison with a true aneurysm, where all vessel layers are intact but dilated and thinned. There are various described causes for SAP – pancreatitis (mostly chronic, as suggested in some articles [3]), blunt abdominal trauma, peptic ulcer disease and iatrogenic injury to the splenic artery or postoperative complications. The mean age of the patients diagnosed with SAP was 51.2 years (35–78 years) in the Tessier *et al.* study [3]. The theory of pancreatitis-induced SAP is based on necrotizing arteritis that destroys an arterial wall and leads to the fragmentation of elastic tissues due to pancreatic enzymes. In trauma, SAP is often intraparenchymal, and involvement of the main splenic artery is uncommon [4]. There is another name for SAP larger than 5 cm – giant SAP.

VAPA has a higher risk for unconcealed rupture compared with a true aneurysm. Hence, VAPA is typically treated the moment it has been diagnosed. Depending upon the location and diameter, pseudoaneurysm rupture is associated with a mortality rate ranging from 25 to 70% [5].

In the majority of the cases, the patients with SAP present to the hospital with a rupture and symptoms of active intraabdominal haemorrhage, very often haemodynamically unstable. Otherwise, SAP is asymptomatic as other VAPAs.

CT is a modality of choice for abdominal assessment with suspected intraabdominal bleeding, splenic trauma, pancreatitis and perforated viscus. It is worth mentioning that ruptured splenic intraparenchymal pseudoaneurysms do not increase in size in delayed phases but look similar to active bleeding on an initial scan (arterial phase) [4]. It is almost impossible to distinguish intraparenchymal SAP from arteriovenous fistula if the latter is one of the differential diagnoses.

VAPAs demonstrate relatively rapid growth rates associated with an early intervention regardless of size [6]. The rate of rupture at presentation to the hospital is noted to be significantly higher in VAPAs than in true aneurysms (76.3 vs. 3.1%) [7]. SAPs and splenic true aneurysms in women who are either pregnant or of childbearing age should be treated regardless of size [8].

Ruptured SAP must be treated as an urgent, life-threatening surgical condition. Regarding the treatment of non-ruptured SAPAs, Society for Vascular Surgery recommends surgery for any size of SAPA because of the possibility of rupture.
